# Pro- and Anti-oxidant Properties of Redox-Active Catechol-Chitosan Films

**DOI:** 10.3389/fchem.2019.00541

**Published:** 2019-07-30

**Authors:** Eunkyoung Kim, Mijeong Kang, Huan Liu, Chunhua Cao, Changsheng Liu, William E. Bentley, Xue Qu, Gregory F. Payne

**Affiliations:** ^1^Institute for Bioscience and Biotechnology Research, University of Maryland, College Park, MD, United States; ^2^Key Laboratory for Ultrafine Materials of Ministry of Education, The State Key Laboratory of Bioreactor Engineering, East China University of Science and Technology, Shanghai, China; ^3^Key Laboratory of Optoelectronic Chemical Materials and Devices, Ministry of Education, School of Chemical and Environmental Engineering, Jianghan University, Wuhan, China

**Keywords:** chitosan, catechol, redox, radical scavenger activity, antimicrobial dressing

## Abstract

Catechols are abundant in nature and are believed to perform diverse biological functions that include photoprotection (e.g., melanins), molecular signaling (e.g., catecholamine neurotransmitters), and mechanical adhesion (e.g., mussel glue). Currently, the structure-property-function relationships for catechols remain poorly resolved, and this is especially true for redox-based properties (e.g., antioxidant, pro-oxidant, and radical scavenging activities). Importantly, there are few characterization methods available to probe the redox properties of materials. In this review, we focus on recent studies with redox-active catechol-chitosan films. First, we describe film fabrication methods to oxidatively-graft catechols to chitosan through chemical, enzymatic, or electrochemical methods. Second, we discuss a new experimental characterization method to probe the redox properties of catechol-functionalized materials. This mediated electrochemical probing (MEP) method probes the redox-activities of catechol-chitosan films by: (i) employing diffusible mediators to shuttle electrons between the electrode and grafted catechols; (ii) imposing tailored sequences of input voltages to “tune” redox probing; and (iii) analyzing the output current response characteristics to infer properties. Finally, we demonstrate that the redox properties of catechol-chitosan films enable them to perform antioxidant radical scavenging functions, as well as a pro-oxidant (reactive oxygen-generation) antimicrobial functions. In summary, our increasing knowledge of catechol-chitosan films is enabling us to better-understand the functions of catechols in biology as well as enhancing our capabilities to create advanced functional materials.

## Introduction

In nature, catechols are believed to confer diverse functions to materials: the photo and free radical protection of melanins (Seagle et al., 2005; Dadachova and Casadevall, 2008; Panzella et al., 2013); the hardening of the insect cuticle through crosslinking (Andersen, 2010; Sugumaran and Barek, 2016; Whitten and Coates, 2017); the adhesion of the mussel glue to surfaces (Ryu et al., 2015; Waite, 2017); the virulence of fungal pathogens (Jacobson, 2000; Nosanchuk and Casadevall, 2006); and the innate immunity of insects (Christensen et al., 2005; Nakhleh et al., 2017). In many cases, the synthesis of catecholic materials is initiated by oxidative reactions that yield a reactive intermediate (e.g., the tyrosinase-generated of *o*-quinones) that can undergo subsequent non-enzymatic reactions. Often the final product has catecholic moieties that could retain redox activity. An overarching hypothesis of our work is that catecholic materials play important roles in redox biology but these roles are under-appreciated, in part, because of the absence of simple characterization methods. Here, we summarize recent research on the biomimetic oxidative-grafting of catechols onto films of the aminopolysaccharide chitosan. To characterize the redox properties of these catechol-chitosan films, we are developing an electrochemical reverse engineering method which is briefly described. Finally, we discuss two applications that highlight the unique technological capabilities for such redox-active catechol-chitosan films.

## Oxidative Grafting of Catechol to Chitosan Films

**Figure 1:**
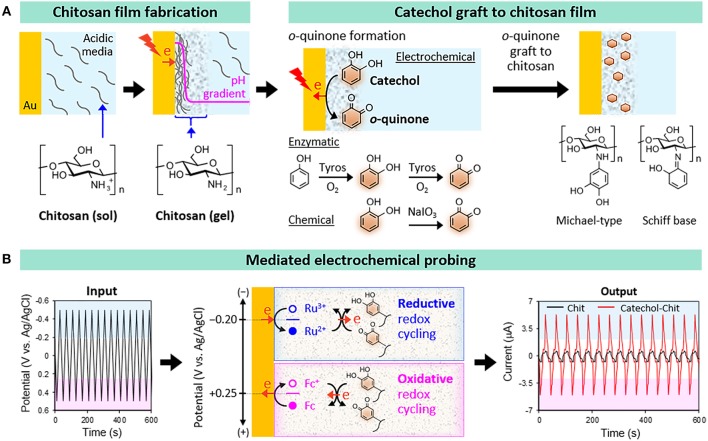
Catechol-chitosan hydrogel films. **(A)** Fabrication by first electrodepositing a film of the aminopolysaccharide chitosan and then oxidatively-grafting catechol moieties to the film. **(B)** Electrochemical reverse engineering approach to characterize the redox properties of the catechol-chitosan films.

Chitosan is a pH-responsive film-forming aminopolysaccharide that has been considered for a variety of diverse applications (Shahidi et al., 1999; Krajewska, 2004; Yi et al., 2005; Peppas et al., 2006; Boateng et al., 2008; Crini and Badot, 2008; Kim et al., 2008; Jayakumar et al., 2011; Shin et al., 2017). As illustrated in Figure 1A, we typically electrodeposit our chitosan films through a cathodic neutralization mechanism in which electrochemical reactions are used to controllably generate a region of high pH that neutralizes the chitosan polymer chains and induces their self-assembly into a thin hydrogel film (Wu et al., 2002; Redepenning et al., 2003). The electrodeposited chitosan film is stable in the absence of an applied voltage provided the pH is retained above about 6.5 (chitosan can re-dissolve at lower pH). In a second step, catechol can be oxidatively-grafted to chitosan using various chemical, electrochemical (Wu et al., 2005, 2006), or enzymatic (Sun et al., 1992; Muzzarelli et al., 1994) approaches. In all these fabrication approaches the catechol is oxidized to a reactive *o*-quinone intermediate that grafts to the chitosan presumably through Schiff-base or Michael-type adduct formation. In summary, chitosan was selected as our film because it: offers appropriate material properties for a range of applications (e.g., biomedical applications); possesses stimuli-responsive film-forming properties that enable electrodeposition; and has nucleophilic primary amines that facilitate catechol's oxidative grafting.

## Characterization of Film Redox Properties

The conventional approach for characterizing materials is to determine chemical structure and evaluate properties to enable the development of the structure-property relations needed to understand the function of a material in nature or to design a material for a technological application. It has been difficult to apply this conventional approach to catechol-based materials (e.g., melanins and polydopamines) (Lee et al., 2007; D'Ischia et al., 2014; Delparastan et al., 2019) for a couple reasons. First, catechol-based materials are often insoluble in water and most solvents, and may have complex non-homogeneous chemical structures that are difficult to resolve using standard chemical characterization methods. For instance, the oxidatively grafted catechols in Figure 1A may be connected to chitosan through various linkages (e.g., Schiff-bases or Michael-type adducts), may engage in the crosslinking of chitosan chains, and/or may involve the grafting of oligomeric-phenolic species to chitosan. Thus, the catechol-chitosan films likely possess complex structures consistent with complex structures of natural catecholic materials (e.g., melanins).

A second reason why it has been difficult to generate structure-property relations for catecholic materials is that important functions (e.g., antioxidant protection and radical scavenging) often rely on redox-properties and the methods for characterizing redox properties of insoluble materials are not particularly versatile. For our example, initial studies showed that our catechol-chitosan films are non-conducting and could not directly exchange electrons with the underlying electrode (Kim et al., 2010). Presumably, these films are non-conducting because the oxidative grafting of catechol does not generate the conjugated aromaticity that is common to conducting polymers. Further, presumably few of the grafted catechol moieties in these hydrogel films (≈1 μm thick) are contacting the underlying electrode which is needed for direct electron exchange. Although these catechol-chitosan films are non-conducting, they have been shown to be redox-active as the grafted catechol moieties can accept electrons from diffusible reductants and donate electrons to diffusible oxidants. Typical approaches to characterize redox-active but non-conducting materials involve incubation of the material in the presence of a diffusible reductant (or oxidant) and measuring how much of this diffusible species is consumed by donating electrons to (or accepting electrons from) the material. Such methods have significant limitations: the methods can be time-consuming; redox activities are typically observed in one direction (oxidation or reduction); and the methods cannot easily determine if the redox reactions are reversible or irreversible (conventional approaches would require multiple solution exchanges to provide the oxidative and reductive conditions to test for the reversibility of redox reactions).

To overcome these limitations, we developed an electrochemical reverse engineering approach that does not require knowledge of chemical structure and focuses on redox properties (Kim et al., 2013b, 2014b; Kang et al., 2018). Specifically, Figure 1B shows that this mediated electrochemical probing (MEP) method: (i) uses electrochemical instrumentation to expose our catechol-chitosan films to controlled voltage inputs; (ii) uses diffusible redox mediators to “transmit” these voltage inputs from the electrode surface into the film; and (iii) generates an output response-current that can be analyzed to assess the film's redox properties.

Experimentally, an electrode coated with the catechol-chitosan film is immersed in a solution containing diffusible mediators that serve to shuttle electrons between the electrode and the grafted catechols. The mediators are molecules that can readily and reversibly donate and accept electrons. Such mediators can be selected from those commonly used in electrochemistry, or can be redox-active molecules derived from biology. Importantly, multiple mediators can be added to the same solution, but each mediator is only “activated” to donate/accept electrons when the voltage of the underlying electrode is poised near this mediator's redox potential (*E*^0^). [Note: this is analogous to a solution containing numerous acid and base species, and an individual species only donates/accepts a proton when the pH is near its pKa].

We initiate redox probing by imposing a tailored sequence of input voltages that serve to “tune” the voltage window being probed. In the example illustrated in Figure 1B, we probed the catechol-chitosan film using two mediators and a cyclic imposed electrode voltage. When the imposed voltage is cycled into the reducing region, the Ru(NH_3_)_6_Cl_3_ mediator (Ru^3+^) is reduced at the electrode to its Ru^2+^ state which diffuses into the film and can donate electrons to the film presumably converting the oxidized quinone moieties into reduced catechol moieties. As illustrated in Figure 1B, this Ru^3+^-mediated transfer of electrons from the electrode to the film involves a reductive redox-cycling mechanism. When the imposed voltage is cycled into the oxidative range, the ferrocene dimethanol mediator (Fc) can engage in oxidative redox-cycling that serves to transfer electrons from the film to the electrode presumably converting the reduced catechol moieties into oxidized quinone moieties. [Note: the Ru^3+^ and Fc mediators engage in one-electron transfer steps while the interconversion between quinone and catechol involves two electrons and may proceed through a semiquinone intermediate].

The output response-current in Figure 1B shows the response-characteristics of the catechol-chitosan film in comparison to those for a control (redox-inactive) chitosan film. One obvious feature of this response is the significant amplification of the Ru^3+^-reduction and Fc-oxidation currents. Amplification of mediator currents is a signature indicating that the catechol-chitosan films are redox-active and can accept electrons (through Ru^3+^-mediated reductive redox-cycling) and donate electrons (through Fc^+^-mediated oxidative redox-cycling). A more subtle point is that these results suggest that the *E*^0^ values for the Ru^3+^ (−0.2 V vs. Ag/AgCl) and Fc^+^ (+0.25 V) mediators bracket the *E*^0^ value for catechol-chitosan film. This *E*^0^ range (−0.2 to +0.25 V vs. Ag/AgCl) is consistent with the *E*^0^ value for reported for catechol (+0.2 V vs. Ag/AgCl) (Behera et al., 2008). A second feature of the response-characteristic is obvious from the input-output curves which show a steady output pattern over multiple cycles. This steady output-response indicates that the catechol-chitosan film can be repeatedly oxidized and reduced.

In summary, the MEP enabled us to determine that the catechol-chitosan film is redox-active and can be repeatedly oxidized and reduced. This ability to accept, store and donate electrons essentially means the catechol-chitosan film is a redox-capacitor (Kim et al., 2012, 2013a, 2014a, 2015; Liu et al., 2014). Importantly, the redox potential of the catechol-chitosan film is in the mid-physiological range and other studies have shown that this film has a broad “substrate range” in that it can accept electrons from various biological reductants (e.g., NADPH and ascorbate) and donate electrons to biological oxidants (e.g., O_2_) (Kim et al., 2012, 2014a). In the following, we cite two applications in which the catechol-chitosan redox-capacitor film can engage in biologically-relevant redox-based “communication.”

## Antioxidant Radical Scavenging Films

In nature, melanins are believed to perform protective functions that are due, in some cases, to their ability to scavenge free radicals (e.g., radiation-induced free radicals). One of the most extreme examples of such protection is suggested by the ability of melanized fungi to survive in the highly radioactive Chernobyl site (Dadachova and Casadevall, 2008; Dighton et al., 2008; Gessler et al., 2014). We used our MEP system to test the hypothesis that the catechol-chitosan film could donate electrons to scavenge (i.e., quench) oxidative free radicals.

**Figure 2:**
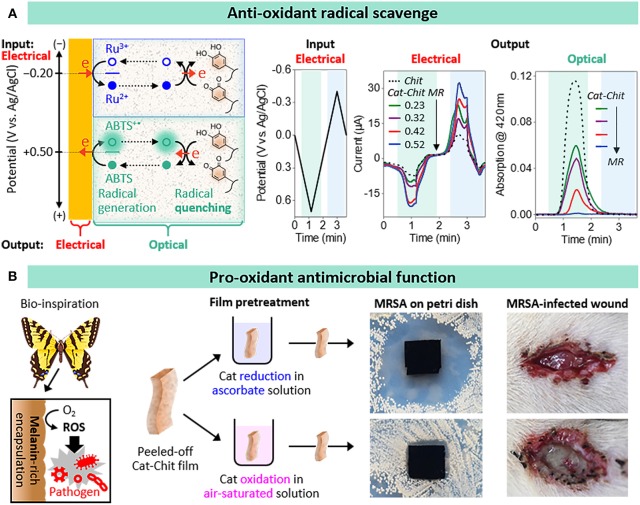
Anti- and pro- oxidant functions of catechol-chitosan films. **(A)** Antioxidant radical scavenging activities: ABTS oxidative redox-cycling associated with radical scavenging is apparent from the amplified electrochemical (electrical) currents and attenuated optical absorbance (results from Cao et al., 2018). **(B)** Bio-inspired pro-oxidant activities associated with reactive oxygen species (ROS) generation confer antimicrobial activities to wound dressings (results from Liu et al., 2018).

To test this radical-scavenging hypothesis, we prepared catechol-chitosan films with different extents of catechol modification (Cao et al., 2018). We used electrochemical measurements to provide a semi-quantitative estimate of film's modification ratio (MR); ratio of grafted catechol moieties per chitosan amine (MRs = 0.23, 0.32, 0.42, and 0.52). Experimentally, we tested for radical-scavenging activity using the standard reagent 2,2′-azino-bis(3-ethylbenzothiazoline-6-sulfonic acid) diammonium salt (ABTS) that can be electrochemically oxidized to generate the ABTS^+•^ radical. As illustrated in Figure 2A we used cyclic voltage inputs to oxidize ABTS for radical generation and used two output measurements to provide evidence that the catechol-chitosan films can quench the ABTS^+•^ radicals by donating electrons through a redox-cycling mechanism. One output measurement was current, and we observed that ABTS oxidation currents were amplified for catechol-modified films compared to unmodified chitosan films, and the extent of amplification increased with the extent of catechol-modification.

The second output measurement was absorbance at 420 nm: the ABTS^+•^ radical solution is green-colored while the ABTS solution is colorless. As illustrated in Figure 2A, when the imposed voltage is cycled into the oxidative region, the absorbance increases consistent with the generation of the ABTS^+•^ radical. For the case, of the unmodified chitosan film, the absorbance is observed to reach a peak and then decrease when the voltage was cycled into the reducing region which is consistent with the electrochemical reduction of the ABTS^+•^ radical. In comparison to the unmodified chitosan film, Figure 2A shows that the catechol-chitosan films attenuated the optical absorbance with the extent of attenuation increasing with the extent of catechol modification.

In summary, these results demonstrate that the catechol-chitosan film can accept electrons to quench the oxidative ABTS^+•^ radical (Cao et al., 2018). We observed this radical scavenging activity is context dependent: the films must be in a reduced state to have available electrons to donate to the ABTS^+•^ radical (this is why Ru^3+^ is included in the electrochemical experiment illustrated in Figure 2A). Importantly, the films can be poised in their reduced state using non-electrochemical reductants such as the inexpensive biological reductant, ascorbic acid (i.e., vitamin C). In additional studies, we observed that the catechol-chitosan film can not only donate electrons to scavenge an oxidative free radical (i.e., ABTS^+•^) but it can also accept electrons to quench a reductive free radical (e.g., paraquat) (Cao et al., 2018). We envision such catechol-chitosan films could be used as protective coating for diverse applications (e.g., clothing).

## Pro-oxidant Reactive Oxygen Generating Activity

While the above example illustrates that the electron donating activities of the catechol-chitosan film enable it to perform protective antioxidant functions, these same redox-activities enable the film to perform pro-oxidant functions. Specifically, Figure 2B illustrates that electron-donation to O_2_ could generate damaging reactive oxygen species (ROS). In fact, is was suggested that the melanin that is synthesized by insects to encapsulate invading pathogenic bacteria (Christensen et al., 2005; Falabella et al., 2012; Nakhleh et al., 2017) may be redox-active and capable of transferring electrons from endogenous reducing equivalents to O_2_ for the sustained and localized generation of antimicrobial ROS (Nappi and Christensen, 2005). Inspired by this example, we prepared free-standing catechol-chitosan films, and converted them to either an oxidized state (by a 2-h exposure to water while pure O_2_ was being bubbled through the water) or to a reduced state (by a 15-min incubation in a 300 mM ascorbic acid solution), and then evaluated their antimicrobial activities (Liu et al., 2018). Specifically, we performed antimicrobial studies using methicillin resistant *Staphylococcus aureus* (MRSA). The photograph of the Petri dish in Figure 2B shows a large zone of inhibition for the catechol-chitosan film that was poised in a reduced state, while little or no inhibition is observed for the catechol-chitosan film that was poised in an oxidized state or for the unmodified chitosan film. [Chemical evidence for H_2_O_2_-generation by the reduced film is provided in the original paper along with evidence that intermittent ascorbate treatments could be used to regenerate the ROS-generating abilities of the catechol-chitosan film (Liu et al., 2018)].

One application for a film that offers localized and prolonged ROS-generating activities is as an antimicrobial wound dressing. Initial studies to test for *in vivo* antimicrobial activities involved an infected wound model using Sprague Dawley (SD) rats. Specifically, an incision was pre-seeded with MRSA, treated with a film (i.e., model dressing) and after 3 days inspected. The photographs in Figure 2B show the wound treated with a catechol-chitosan film that had been poised in its reduced state (by ascorbic acid treatment) showed no obvious festering compared to the control catechol-chitosan film that had been poised in an oxidized state. After taking these photographs, the films were recovered and analyzed, and we observed a 2 order-of-magnitude lower bacterial concentration for the reduced catechol-chitosan film compared to the control films (Liu et al., 2018).

## Conclusions

In summary, the oxidative grafting of catechols to chitosan confers redox activities that can be used to perform either protective antioxidant radical-scavenging functions, or pro-oxidant antimicrobial functions. Importantly, these results indicate that catecholics are active materials and display context-dependent behaviors (oxidant vs. reductant) that could have important implications in biology and technology.

## Author Contributions

EK developed fabrication and electrochemical reverse engineering methodologies. MK developed spectroelectrochemical reverse engineering methodology. HL performed experiments demonstrating antimicrobial properties and wound healing capabilities. CC performed experiments demonstrating radical scavenging activities. CL provided insights on requirements for biomedical materials for wound healing. WB provided insights on the use of mediated electrochemical probing in biological systems. XQ coordinated research on the antimicrobial wound dressing. GP coordinated research on the radical scavenging films and prepared the manuscript.

### Conflict of Interest Statement

The authors declare that the research was conducted in the absence of any commercial or financial relationships that could be construed as a potential conflict of interest.
